# Enhanced Levels of Interleukin-8 Are Associated with Hepatitis B Virus Infection and Resistance to Interferon-Alpha Therapy

**DOI:** 10.3390/ijms151121286

**Published:** 2014-11-17

**Authors:** Kai Yang, Shi-He Guan, Hao Zhang, Ying Pan, Yuan-Yuan Wu, Ai-Hua Wang, Bei-Bei Sun

**Affiliations:** Department of Laboratory Medicine, Second Affiliated Hospital of Anhui Medical University, Hefei 230601, China; E-Mails: ykmedicine@163.com (K.Y.); haozhang595@163.com (H.Z.); pany7876@163.com (Y.P.); wuyy6728@163.com (Y.-Y.W.); 314wcy@163.com (A.-H.W.); sunbeibei87816@163.com (B.-B.S.)

**Keywords:** interleukin-8, interferon-alpha, chronic hepatitis B, inflammation, fibrosis

## Abstract

The objective of this study was to analyze the expression levels of IL-8 in serum and liver tissues from patients with chronic hepatitis B (CHB) infection and to investigate whether IL-8 may antagonize interferon-alpha (IFN-α) antiviral activity against HBV. IL-8 expression in serum was determined by enzyme linked immunosorbent assay (ELISA), and fluorescence-based quantitative real-time PCR (RT-qPCR) was used to measure IL-8 mRNA in peripheral blood mononuclear cells (PBMCs) in patients with CHB. IL-8 protein expression was detected in liver biopsy tissues by immunohistochemistry. In addition, the differences in serum IL-8 and PBMCs mRNA levels were also observed in patients with different anti-viral responses to IFN-α. Compared to normal controls, serum IL-8 protein and mRNA levels were increased in CHB patients, IL-8 levels were positively correlated with the severity of liver inflammation/fibrosis. Moreover, serum IL-8 protein and mRNA levels were positively correlated with serum alanine aminotransferase (ALT) level and negatively correlated with serum prealbumin (PA) level. IL-8 expression was mainly located in portal area of liver tissues and was increased with the severity of liver inflammation and fibrosis stage. The expression serum and mRNA levels of IL-8 in the CHB patients with a complete response to IFN-α are significantly lower than that of the patients with non-response to IFN-α treatment. It is suggested that IL-8 might play important roles in the pathogenesis of CHB. Moreover, interferon resistance may be related to the up-regulation of IL-8 expression in the patients did not respond to IFN-α treatment.

## 1. Introduction

Chronic hepatitis B (CHB) virus infection is a significant problem of public health with about 400 million hepatitis B virus (HBV) carriers worldwide [1]. CHB carries a substantial risk to progress towards liver fibrosis, cirrhosis, and hepatocellular carcinoma [2,3,4]. Therefore, it is particularly important for drug treatment of CHB to suppress HBV replication steadily and delay the progression of liver pathological process. Interferon-alpha (IFN-α) and its derivatives are the mainstay of treatment for chronic HBV infection [5,6,7,8]. In clinical practice, some patients show low or no response to IFN treatment, known as “interferon resistance” [9]. Recently, it has been revealed that the virus induces expression of the proinflammatory chemokine IL-8 to partially inhibit the antiviral actions of IFN-α *in vitro* [10,11,12].

IL-8 is a member of the cysteine-x-cysteine (CXC) chemokine subfamily and is produced by blood cells and many types of tissues [13]. Several observations suggested that IL-8 might be involved in the pathogensis of chronic liver disease [14,15]. For instance, IL-8 is induced in response to expression of nonstructural protein 5A (NS5A) protein of hepatitis C virus [16]. In another study, it was shown that the X protein of HBV (HBx) transactivates IL-8 expression through NF-κB and CCAAT enhancer-binding protein (C/EBP)-like *cis*-elements [17]. IL-8 has been used as a marker of liver damage for diagnosis of various types hepatitis virus infection [18,19]; however, the relationship between levels of IL-8, HBV infection, and biochemical response to IFN-α remain unclear.

In the present study, we observed the serum IL-8 protein levels, IL-8 mRNA levels and IL-8 protein expression in hepatocytes of patients with untreated chronic HBV infection and explored the relationship between IL-8 expression and the severity of liver inflammation/fibrosis. The differences of IL-8 expression in serum and mRNA in peripheral blood mononuclear cells (PBMCs) of patients with CHB were observed, in order to investigate whether IL-8 involves in the resistance of HBV to IFN-α.

## 2. Results

### 2.1. Serum IL-8 Protein and mRNA Were Augmented in Chronic Hepatitis B (CHB) Patients

**Figure 1:**
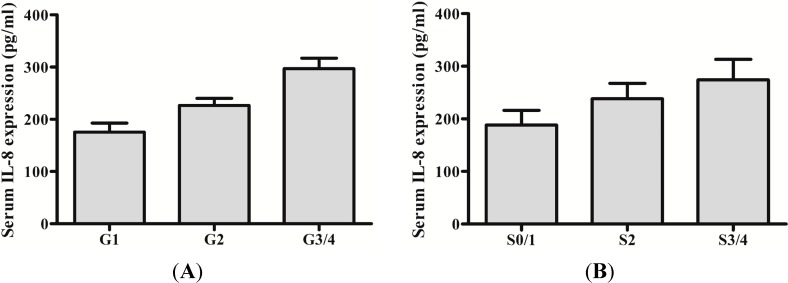
Serum expression of IL-8 in chronic hepatitis B (CHB) with different hepatic inflammation stages or fibrosis grades. (**A**) Detection of IL-8 protein in serum of patients with different hepatic inflammation stages by enzyme linked immunosorbent assay (ELISA); (**B**) Detection of IL-8 protein using ELISA in serum of patients with different grades of hepatic fibrosis.

**Figure 2:**
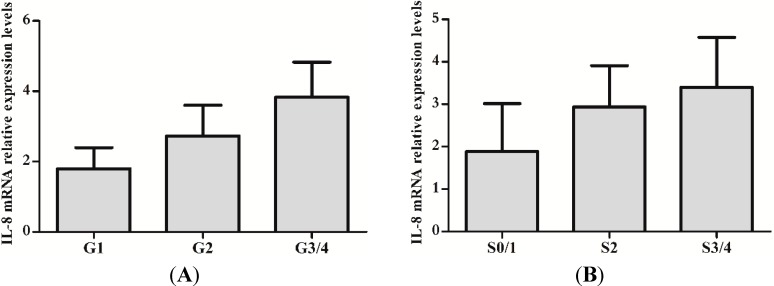
mRNA expression of IL-8 in Peripheral Blood Mononuclear Cells (PBMCs) of CHB with different hepatic inflammation stages or fibrosis grades. (**A**) Detection of IL-8 mRNA in PBMCs of CHB with different hepatic inflammation stages by RT-qPCR; (**B**) Detection of IL-8 mRNA in PBMCs of CHB with different hepatic hepatic fibrosis grades by RT-qPCR.

The clinical data of 66 CHB patients is shown in Table 1. To evaluate whether IL-8 protein and mRNA were increased in CHB, the levels of IL-8 protein and mRNA between CHB patients and health individuals were analyzed. As expected, IL-8 protein and mRNA were significant higher in CHB patients than that of health individuals (217.971 ± 48.570 *vs.* 143.821 ± 26.968, *t* = 8.464, *p* < 0.01) (1.329 ± 0.079 *vs.* 2.435 ± 1.296, *t* = 5.037, *p* < 0.01). We also investigated whether protein and mRNA levels of IL-8 were increased with severity of liver inflammation and fibrosis. Both protein and mRNA levels of IL-8 in inflammation grade 3/4 were significantly increased compared with that of inflammation grade 1/2 (*p* < 0.01) (Figure 1A and Figure 2A). Likewise, fibrosis stage 3/4 levels were higher than that of fibrosis stage 0/1/2 (*p* < 0.01) (Figure 1B and Figure 2B).

**Table 1:** Complications and laboratory tests in patients with hepatitis B virus (HBV) (mean ± SD, *n* = 66).

Characteristic	Patients (*n* = 66)
Gender (M/F)	29/37
Age (Years)	42.71 ± 13.52
Liver inflammation Grade (1, 2, 3, 4)	29/24/9/4
Liver fibrosis stage (0, 1, 2, 3, 4)	39/10/5/12
ALT (Units/L)	100.28 ± 32.44
PA (mg/L)	217.97 ± 48.57
HBV DNA (log_10_ copies per mL)	5.73 ± 1.95

### 2.2. IL-8 Expression in Liver Tissues of CHB Patients

**Figure 3:**
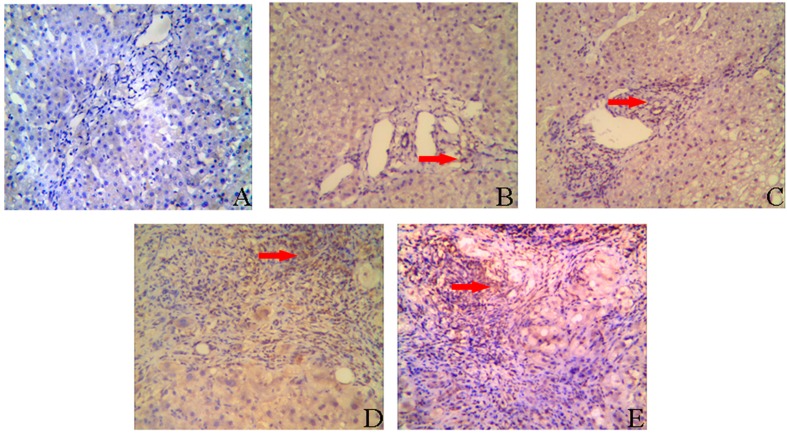
Immunohistochemical staining for IL-8. IL-8 protein expression was evaluated by a streptavidin-perosidase (SP) immunohistochemical method (magnification ×200). The hepatocytes had weak expression of IL-8. IL-8 protein expression was mainly localized in the portal area. (**A**) Liver tissue of normal controls; (**B**) Liver tissue of CHB with necroinflammation grades 1 and fibrosis stages 1 (G1S1); (**C**) Liver tissue of CHB with necroinflammation grades 2 and fibrosis stages 2 (G2S2); (**D**) Liver tissue of CHB with necroinflammation grades 3 and fibrosis stages 3 (G3S3); (**E**) Liver tissue of CHB with necroinflammation grades 4 and fibrosis stages 4 (G4S4). The red arrow indicates IL-8 protein expression in the portal area.

Immunohistochemistry was also performed to detect the expression of IL-8 in the CHB patients. IL-8 was weakly expressed in hepatocytes and mainly visualized in the portal area (Figure 3B–E). IL-8 in normal liver tissues nearly undetectable (Figure 3A). The IL-8 expression in the portal area positively correlated with inflammation grade (*r* = 0.660, *p* < 0.001) and fibrosis stage (*r* = 0.698, *p* < 0.001) (Table 2 and Table 3).

**Table 2:** The relationship between the expression of IL-8 and the degree of liver inflammation, *r* = 0.660, *p* < 0.001.

Inflammation Stages	Cases	Expression of IL-8			
		−	+	++	+++
G1	29	6	19	4	0
G2	24	1	4	15	4
G3/4	13	0	0	7	6

**Table 3:** The relationship between the expression of IL-8 and the degree of liver fibrosis, *r* = 0.698, *p* < 0.001.

Fibrosis Grades	Cases	Expression of IL-8			
		−	+	++	+++
S0/1	39	22	17	0	0
S2	10	2	5	3	0
S3/4	17	0	2	6	9

### 2.3. IL-8 Expression Is Positively Related with Serum Alanine Aminotransferase (ALT) Level in CHB Patients

**Figure 4:**
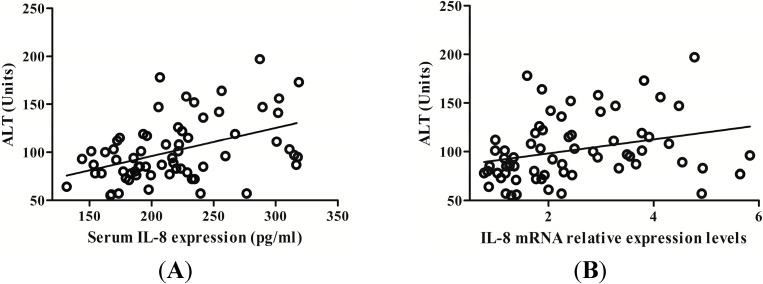
The correlation between ALT levels and IL-8 expression. (**A**) Serum IL-8 protein was positively correlated with serum alanine aminotransferase (ALT); (**B**) PBMCs IL-8 mRNA was also positively correlated with serum ALT levels.

Serum alanine aminotransferase (ALT) has been widely recognized as an important marker of inflammation in liver disease. We assessed the correlation between serum ALT, IL-8 protein values and mRNA levels, respectively. Results showed that serum ALT level was progressively augmented along with the increase of serum IL-8 level and there was positive correlation between serum IL-8 and ALT level (*r* = 0.419, *p* < 0.001) (Figure 4A). Additionally, IL-8 mRNA levels in PBMCs was positively correlated with ALT level (*r* = 0.348, *p* = 0.004) (Figure 4B).

### 2.4. IL-8 Expression Was Negatively Correlated with Serum Prealbumin (PA) Level

**Figure 5:**
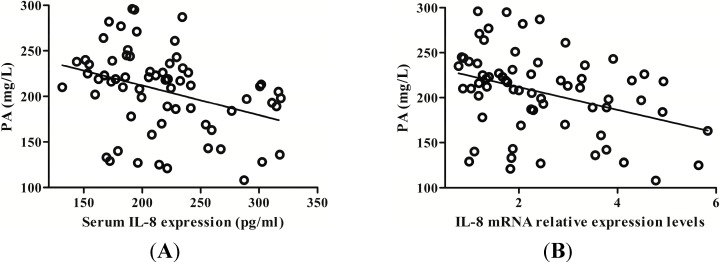
The correlation between prealbumin (PA) levels and IL-8 expression. (**A**) Serum IL-8 protein was negatively correlated with serum PA; (**B**) PBMCs IL-8 mRNA was also negatively correlated with serum PA levels.

An altered liver function may impair prealbumin (PA) synthesis. Clinical analysis revealed that serum PA level gradually decreased with the increase of hepatic IL-8 expression. Serum IL-8 expression was inversely proportional to serum PA level (*r* = 0.384, *p* = 0.001) (Figure 5A). In addition, IL-8 mRNA in PBMCs was also negatively correlated with serum PA level (*r* = 0.355, *p* = 0.003) (Figure 5B).

### 2.5. Levels of IL-8 in Serum and the Peripheral Blood Mononuclear Cells (PBMCs) Responses to Interferon-Alpha (IFN-α) Therapy

**Figure 6:**
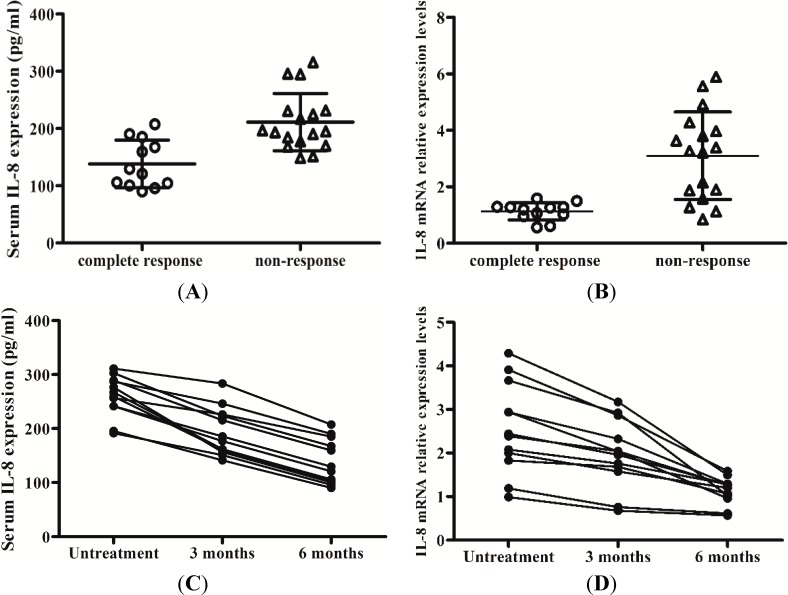

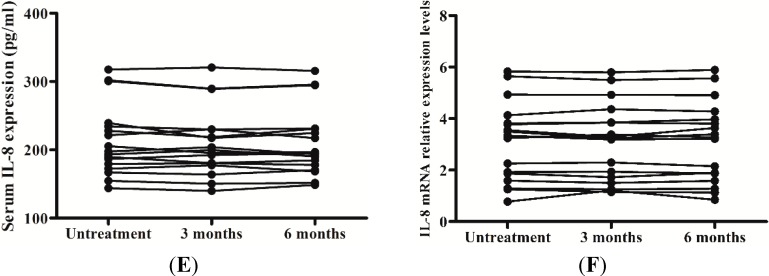
Expression of IL-8 in serum and PBMCs of patients who had a complete response compared to patients who did not respond to antiviral therapy by interferon-alpha (IFN-α) during treatment. (**A**) Detection of serum IL-8 of the patients who had complete response *vs.* no response to antiviral therapy by IFN-α after treatment by ELISA; (**B**) Detection of IL-8 mRNA in PBMCs of the patients who had complete response *vs.* no response to antiviral therapy by IFN-α after treatment by RT-qPCR; (**C**) Detection of serum IL-8 of the patients who had complete response to antiviral therapy by IFN-α during treatment by ELISA; (**D**) Detection of IL-8 mRNA in PBMCs of the patients who had complete response to antiviral therapy by IFN-α during treatment by RT-qPCR; (**E**) Detection of serum IL-8 of the patients who had no response to antiviral therapy by IFN-α during by ELISA; (**F**) Detection of IL-8 mRNA in PBMCs of the patients who had no response to antiviral therapy by IFN-α during treatment by RT-qPCR.

After treatment with IFN-α, the results of ELISA and RT-qPCR showed that CHB patients with a complete response to antiviral therapy by IFN-α had a lower expression of IL-8 in the serum and PBMCs. Meanwhile, the expression levels of IL-8 in the serum and PBMCs of CHB patients without response to IFN-α were significantly higher (138.076 ± 41.665 *vs.* 211.039 ± 50.016, *t* = 4.271, *p* < 0.01) (1.133 ± 0.311 *vs.* 3.099 ± 1.553, *t* = 4.304, *p* < 0.01) (Figure 6A,B).

**Figure 7:**
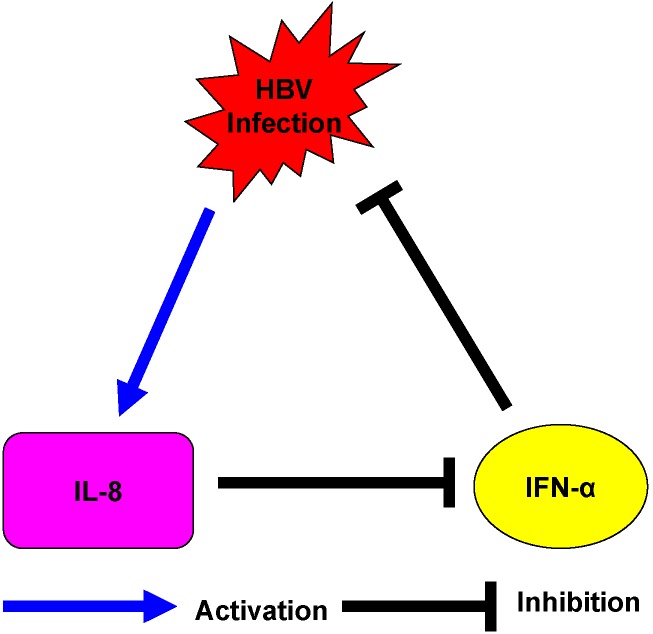
Schematic diagram. Hepatitis B virus (HBV) infection upregulates IL-8 expression in hepatocytes and PBMCs, which might inhibit antiviral action of IFN-α on HBV.

During treatment with IFN-α, IL-8 serum levels and mRNA levels in CHB patients with a complete response to therapy were decreased, while the changes of IL-8 in the serum and PBMCs of the patients without response to IFN-α were not obviously (Figure 6C–F). This indicated that HBV induces the expression of IL-8 might attenuate the antiviral activity of IFN-α (Figure 7).

## 3. Discussion

In this study, we characterized the expression levels of IL-8 during chronic hepatitis B infection. Consistent with previous studies [20,21], we confirmed that there is a significant increase in the expression of IL-8 in the serum and liver tissues of HBV-infected patients.

IL-8 exerts proinflammatory effects in various cell types [22,23,24]. Moreover, it is has been reported that HBV infection activates the expression of IL-29, IL-8, and cyclooxygenase-2 (COX-2) by an unrecognized mechanism [21]. Our present study showed that a significant correlation between IL-8 expression in serum and liver tissue and the stage of liver inflammation. As the severity of liver inflammation got higher, IL-8 levels increased gradually. This is probably because IL-8 is closely related to NK cells that activates a variety of immune cells to release inflammatory mediators, leading to repeated inflammation and damage of liver function [25]. This is also the reason why serum and hepatic IL-8 expression are positively correlated with serum ALT and negatively correlated with PA levels. This further indicated that IL-8 played important roles in liver injury of chronic hepatitis B. We also observed a significant association between serum and liver tissues IL-8 expression with grade of liver fibrosis. This is consistent with the findings of several recent studies reporting that IL-8 contributes to establish a profibrogenic microenvironment in chronic liver disease [13].

IFN-α is a cytokine drug and has been proven to be effective in the treatment of chronic hepatitis B patients. Unfortunately, when patients suffering from chronic hepatitis B are treated with IFN-α, only 30%–40% show clearance of HBV serum markers and normalization of liver function. This is likely related to interferon resistance existing in the patients’ bodies [26]. The exact mechanism of the interferon resistance remains unclear. The accumulating evidence indicates that IL-8 induced by viruses may contribute to counteract IFN-α antiviral action. As such, IL-8 plays a negative role in antiviral activity of IFN-α and an inverse correlation has been found between serum IL-8 levels and sensitivity to IFN-α therapy among patients infected with hepatitis C virus [27]. In our study, IL-8 levels in serum and PBMCs of patients with a complete response were significantly lower than the nonresponders. It is suggested that IL-8 may contribute to reduce HBV sensitivity to IFN-α, and that IFN-α therapy reduces the expression of IL-8. This provides important clue for the treatment of hepatitis B with IFN-α in clinical practice.

Our study has several limitations. Most importantly, clinical association studies cannot prove causal relationship. The correlation between hepatitis B virus e antigen (HBeAg)/HBx presentation and IL-8 expression therefore remain elusive in our study. It has been demonstrated that IL-8 expression was induced by HBx in hepatocytes while down-regulated in HBeAg-positive Hepatoblastoma G2 (HepG2) cells. Recently, HBeAg-negative patients had been reported to have significantly higher levels of IL-8 than HBeAg-positive patients [28]. However, we found a slightly increased IL-8 levels in HBeAg-negative patients, which had no statistical difference compared with that in HBeAg-positive patients. In this regard, the discrepancy between ours results and others documented may be due to the variations in viral subtypes and host genetic background. Larger studies are necessary to fully address this important question, as well as to assess the correlation between HBx presentation and IL-8 expression.

In summary, our study confirms the enhanced expression of IL-8 in patients with CHB. Furthermore, serum and hepatic IL-8 expression is correlated with the severity of liver inflammation/fibrosis in patients with chronic HBV infection. Our results also showed that IL-8 in serum and PBMCs of CHB patients with a complete response to IFN-α are significantly higher than that of the patients with non-response to IFN-α. It is suggested that IL-8 could be favorable for attenuate the antiviral of IFN-α, which is one of mechanisms of persistent viral infection. Therefore, we believe that IL-8 play an important role in the pathogenesis of CHB.

## 4. Materials and Methods

### 4.1. Patients Population

The Review Board of Second Affiliated Hospital of Anhui Medical University approved this study (approval number: AHMU2H2010-0017; 11 October 2010). Written informed consent was obtained according to the guidance of the Chinese National Ethics Regulation Committee. Sixty-six patients with CHB were selected from the clinic or hospitalization unit at the department of infectious diseases of Second Affiliated Hospital of Anhui Medical University during January 2011 and December 2013. Patients had no history of treatment for HBV prior to the study. The clinical diagnosis of all selected patients complied with the standards for diagnoses of CHB prevention and treatment guidelines recommended by Liver disease of the Chinese medical association. All patients were treated with recombinant human IFN-α-2b (trade name: Andafen, Anhui Anke Biotechnology (Group) Co., Ltd., Hefei, China), which was injected intramuscularly at a dose of 5 million U three times a week for 6 months. Among the total patients, 12 cases had complete response to IFN-α-2b therapy and 17 patients had non-response to IFN-α-2b treatment.

### 4.2. Peripheral Blood Mononuclear Cells (PBMCs) Isolation and Detection of IL-8 mRNA

Using EDTA as an anticoagulant, PBMCs were separated by density gradient centrifugation with a lymphocyte separation medium. Total RNA was prepared from PBMCs using Trizol (Invitrogen, Carlsbad, CA, USA) and quantified by NanoDrop 1000 (NanoDrop Technology, Wilmington, DE, USA) with the ratio of A260/A280 more than 1.8. The reversetranscription was performed using a Revert Aid First Strand cDNA Synthesis Kit (Thermo Scientific, San Jose, CA, USA) according to the manufacturer’s instructions. For the RT-qPCR analysis, aliquots of double-stranded cDNA were amplified using Fast Start Universal SYBR Green Master (ROX) (Roche Diagnostics, Indianapolis, IN, USA). qPCR analysis was performed in an Strata Gene MX3000P Detection System (Stratagene, La Jolla, CA, USA) according to the manufacturer’s instructions. The PCR cycling conditions were as follows: 95 °C for 10 min, followed by 40 cycles of denaturation at 95 °C for 10 s, annealing at 60 °C for 1 min and extension step at 72 °C for 10 s. Quantification cycle (Cq) at which emission rises above baseline were automatically calculated by the RT-qPCR system. Each Cq value was normalized to two reference genes (glyceraldehyde-3-phosphate dehydrogenase (*GAPDH*) and β tubulin). The validation of reference genes have be carried out according to minimum information for publication of quantitative real-time PCR experiments (MIQE) guidelines [29,30]. In addition, we analyzed the melting curve of each PCR product in each PCR session and confirmed that no non-specific products had been produced. Negative controls were checked with samples in which the RNA templates were replaced by nuclease-free water in the reactions. For the each sample, the relative expression level (defined as fold change) of the target gene was determined by the equation 2^−ΔΔ*C*q^ (Δ*C*q = Δ*C*q ^IL-8^ − Δ*C*q ^(GAPDH + β tubulin)/2^; ΔΔ*C*q = Δ*C*q − Δ*C*q ^health individual^). The expression level was normalized to the fold change detected in the corresponding a health individual, which was defined as 1.0. All reactions were performed in triplicate. Primer sequences are listed in Table 4.

**Table 4:** Primers for the RT-qPCR.

Gene	Forward Primer (5'→3'); Reverse Sequence (5'→3')	Amplicon Size	GenBank Acc. No.
*IL-8*	CATACTCCAAACCTTTCCACCCC; TCAGCCCTCTTCAAAAACTTCTCCA	175 bp	NM_000584
*GAPDH*	AAGGCTGTGGGCAAGG; TGGAGGAGTGGGTGTCG	238 bp	NM_001256799
*β Tubulin*	GCACATAGTAGGCGCTCAAT; ATCTGGAGACCCAGCTTCTT	175 bp	NM_178014

### 4.3. Detection of IL-8 in Serum

Peripheral blood samples of patients and normal controls were collect and placed in tubes. After centrifugation at speed of 1000× *g* for 15 min, serum samples were harvested for ELISA. IL-8 levels in serum were determined by ELISA according to the manufacturer’s protocol (R&D Systems Europe Ltd., Abingdon, UK).

### 4.4. Immunostaining Procedures

All biopsy specimen tissues were fixed in 10% formalin and embedded in paraffin. Then, liver tissue section were incubated with anti-IL-8 (1:100) overnight at 4 °C after blocking endogenous peroxidase activity with 0.3% H_2_O_2_. Section were then incubated with goat-anti-rabbit secondary antibodies with PBS, autoradiography was performed with DAB followed by counterstaining with hematoxylin. An intensity score was assigned, representing the average intensity of positive cells (0, none; 1, weak; 2, intermediate; and 3, strong). A proportion score was assigned, which represented the estimated proportion of positive-staining cells (0, <5%; 1, 5%–25%; 2, 26%–50%; 3, 51%–75%; and 4, >75%). The proportion and intensity scores were then added to obtain a total score, which ranged from 0 to 8. Slides were scored by pathologists who did not have knowledge of the ligand-binding results or patient outcomes. The total score was further divided into the score as follows: <2, (−); 2–3, (+); 4–5, (++); 6–7, (+++).

### 4.5. Statistical Analysis

All data were presented as means ± SD. Differences among groups were assessed by using unpaired Student *t* test and one-way analysis of variance (ANOVA). *p* value less than 0.05 was considered to be statistically significant. For determination of correlation between different variables Spearman’s correlation coefficient was used, whenever appropriate. Calculations were performed with the Statistical Product and Service Solutions (SPSS) version 16.0 statistical software package (SPSS Inc., Chicago, IL, USA).

## 5. Conclusions

We reported a significant increase in the expression of IL-8 in the serum and liver tissues of HBV-infected patients. We did not find a correlation between IL-8 and HBeAg presentation. Our present study showed that a significant correlation between serum and liver tissues IL-8 expression and stage of liver inflammation and grade of liver fibrosis. Moreover, serum and hepatic IL-8 expression were significantly positively correlated with serum ALT and negatively related to PA levels. Interestingly, IL-8 levels in the serum and PBMCs of patients with a complete response was significantly lower than the nonresponders. The study results suggest that IL-8 plays an important role in the pathogenesis of CHB.

## References

[1] Akbar S.M., Horiike N., Onji M. (2006). Immune therapy including dendritic cell based therapy in chronic hepatitis B virus infection. World J. Gastroenterol..

[2] Chang T.T., Liaw Y.F., Wu S.S., Schiff E., Han K.H., Lai C.L., Safadi R., Lee S.S., Halota W., Goodman Z. (2010). Long-term entecavir therapy results in the reversal of fibrosis/cirrhosis and continued histological improvement in patients with chronic hepatitis B. Hepatology.

[3] Liaw Y.F. (2013). Reduction of cirrhosis and hepatocellular carcinoma with antiviral therapy in chronic hepatitis B. Hepatology.

[4] Huang X., Hollinger F.B. (2014). Occult hepatitis B virus infection and hepatocellular carcinoma: A systematic review. J. Viral Hepat..

[5] Hansen B.E., Buster E.H., Steyerberg E.W., Lesaffre E., Janssen H.L. (2010). Prediction of the response to peg-interferon-α in patients with HBeAg positive chronic hepatitis B using decline of HBV DNA during treatment. J. Med. Virol..

[6] Kittner J.M., Sprinzl M.F., Grambihler A., Weinmann A., Schattenberg J.M., Galle P.R., Schuchmann M. (2012). Adding pegylated interferon to a current nucleos(t)ide therapy leads to HBsAg seroconversion in a subgroup of patients with chronic hepatitis B. J. Clin. Virol..

[7] Ratnam D., Dev A., Nguyen T., Sundararajan V., Harley H., Cheng W., Lee A., Rusli F., Chen R., Bell S. (2012). Efficacy and tolerability of pegylated interferon-α-2a in chronic hepatitis B: A multicenter clinical experience. J. Gastroenterol. Hepatol..

[8] Senturk H., Baysal B., Tahan V., Zerdali H., Ozaras R., Tabak F., Mert A., Canbakan B., Tabak O., Ozbay G. (2011). Long-term effect of interferon therapy in patients with HBeAg positive chronic hepatitis B infection. Dig. Dis. Sci..

[9] Chen J., Wu M., Zhang X., Zhang W., Zhang Z., Chen L., He J., Zheng Y., Chen C., Wang F. (2013). Hepatitis B virus polymerase impairs interferon-α-induced STA T activation through inhibition of importin-α5 and protein kinase C-δ. Hepatology.

[10] Mihm U., Herrmann E., Sarrazin U., von Wagner M., Kronenberger B., Zeuzem S., Sarrazin C. (2004). Association of serum interleukin-8 with virologic response to antiviral therapy in patients with chronic hepatitis C. J. Hepatol..

[11] Randall R.E., Goodbourn S. (2008). Interferons and viruses: An interplay between induction, signaling, antiviral responses and virus countermeasures. J. Gen. Virol..

[12] Lee C.M., Yen Y.H., Hung C.H., Lu S.N., Wang J.H., Wang J.C., Chen C.H., Kee K.M., Hu T.H., Changchien C.S. (2011). Liver interleukin-8 messenger RNA expression and interferon sensitivity-determining region mutations relate to treatment response in hepatitis C 1b. Antivir. Ther..

[13] Zimmermann H.W., Seidler S., Gassler N., Nattermann J., Luedde T., Trautwein C., Tacke F. (2011). Interleukin-8 is activated in patients with chronic liver diseases and associated with hepatic macrophage accumulation in human liver fibrosis. PLoS One.

[14] Tachibana Y., Nakamoto Y., Mukaida N., Kaneko S. (2007). Intrahepatic interleukin-8 production during disease progression of chronic hepatitis C. Cancer Lett..

[15] Hill D.B., Marsano L.S., McClain C.J. (1993). Increased plasma interleukin-8 concentrations in alcoholic hepatitis. Hepatology.

[16] Girard S., Shalhoub P., Lescure P., Sabile A., Misek D.E., Hanash S., Bréchot C., Beretta L. (2002). An altered cellular response to interferon and up-regulation of interleukin-8 induced by the hepatitis C viral protein NS5A uncovered by microarray analysis. Virology.

[17] Mahé Y, Mukaida N., Kuno K., Akiyama M., Ikeda N., Matsushima K., Murakami S. (1991). Hepatitis B virus X protein transactivates human interleukin-8 gene through acting on nuclear factor κB and CCAAT/enhancer-binding protein-like *cis*-elements. J. Biol. Chem..

[18] Masumoto T., Ohkubo K., Yamamoto K., Ninomiya T., Abe M., Akbar S.M., Michitaka K., Horiike N., Onji M. (1998). Serum IL-8 levels and localization of IL-8 in liver from patients with chronic viral hepatitis. Hepatogastroenterology.

[19] Mahmood S., Sho M., Yasuhara Y., Kawanaka M., Niiyama G., Togawa K., Ito T., Takahashi N., Kinoshita M., Yamada G. (2002). Clinical significance of intrahepatic interleukin-8 in chronic hepatitis C patients. Hepatol. Res..

[20] Wang J.Y., Wang X.L., Liu P. (1999). Detection of serum TNF-α, IFN-β, IL-6 and IL-8 in patients with hepatitis B. World J. Gastroenterol..

[21] Yu Y., Gong R., Mu Y., Chen Y., Zhu C., Sun Z., Chen M., Liu Y., Zhu Y., Wu J. (2011). Hepatitis B virus induces a novel inflammation network involving three inflammatory factors, IL-29, IL-8 and cyclooxygenase-2. J. Immunol..

[22] Hartman Z.C., Poage G.M., den Hollander P., Tsimelzon A., Hill J., Panupinthu N., Zhang Y., Mazumdar A., Hilsenbeck S.G., Mills G.B. (2013). Growth of triple-negative breast cancer cells relies upon coordinate autocrine expression of the proinflammatory cytokines IL-6 and IL-8. Cancer Res..

[23] Bauer M., Gräbsch C., Gminski R., Ollmann A.I., Borm P., Dietz A., Herbarth O., Wichmann G. (2012). Cement-related particles interact with proinflammatory IL-8 chemokine from human primary oropharyngeal mucosa cells and human epithelial lung cancer cell line A549. Environ. Toxicol..

[24] Ganesan S., Unger B.L., Comstock A.T., Angel K.A., Mancuso P., Martinez F.J., Sajjan U.S. (2013). Aberrantly activated EGFR contributes to enhanced IL-8 expression in COPD airways epithelial cells via regulation of nuclear FoxO3A. Thorax.

[25] Dunn C., Brunetto M., Reynolds G., Christophides T., Kennedy P.T., Lampertico P., Das A., Lopes A.R., Borrow P., Williams K. (2007). Cytokines induced during chronic hepatitis B virus infection promote a pathway for NK cell-mediated liver damage. J. Exp. Med..

[26] Chen H., Wang L.W., Huang Y.Q., Gong Z.J. (2010). Interferon-alpha induces high expression of APOBEC3G and STAT-1 *in Vitro* and *in Vivo*. Int. J. Mol. Sci..

[27] Polyak S.J., Khabar K.S., Paschal D.M., Ezelle H.J., Duverlie G., Barber G.N., Levy D.E., Mukaida N., Gretch D.R. (2001). Hepatitis C virus nonstructural 5A protein induces interleukin-8, leading to partial inhibition of the interferon-induced antiviral response. J. Virol..

[28] Pollicino T., Bellinghieri L., Restuccia A., Raffa G., Musolino C., Alibrandi A., Teti D., Raimondo G. (2013). Hepatitis B virus (HBV) induces the expression of interleukin-8 that in turn reduces HBV sensitivity to interferon-alpha. Virology.

[29] Bustin S.A., Benes V., Garson JA., Hellemans J., Huggett J., Kubista M., Mueller R., Nolan T., Pfaffl M.W., Shipley G.L. (2009). The MIQE guidelines: Minimum information for publication of quantitative real-time PCR experiments. Clin. Chem..

[30] Bustin S.A., Beaulieu J.F., Huggett J., Jaggi R., Kibenge F.S., Olsvik P.A., Penning L.C., Toegel S. (2010). MIQE précis: Practical implementation of minimum standard guidelines for fluorescence-based quantitative real-time PCR experiments. BMC Mol. Biol..

